# Essential Oil of *Xylopia frutescens* Controls Rice Sheath Blight Without Harming the Beneficial Biocontrol Agent *Trichoderma asperellum*

**DOI:** 10.3390/plants15010031

**Published:** 2025-12-22

**Authors:** Paulo Ricardo S. Fernandes, Dalmarcia de Souza C. Mourão, Luís O. Viteri, Adauto A. Silva Júnior, Muhammad Bilal, Anila Kanwal, Osmany M. Herrera, Manuel A. Gonzalez, Leandro A. Souza, Ana G. Amaral, Thayse Cavalcante da Rocha, Marcos Paz Saraiva Câmara, Raphael Sanzio Pimenta, Marcos V. Giongo, Eugênio E. Oliveira, Raimundo Wagner S. Aguiar, Gil R. Santos

**Affiliations:** 1 Departamento de Fitopatologia, Universidade Federal do Tocantins, Gurupi 77402-970, TO, Brazil; pauloricardosena@mail.uft.edu.br (P.R.S.F.); dalmarciaadm@uft.edu.br (D.d.S.C.M.); luis.viteri@uft.edu.br (L.O.V.); bilalsaif987@gmail.com (M.B.); kanwalanila46@gmail.com (A.K.); osmany.herrera@mail.uft.edu.br (O.M.H.); manuelgon51295@gmail.com (M.A.G.); engleandroalves2410@gmail.com (L.A.S.); 2 Programa de Pós-Graduação em Produção Vegetal, Universidade Federal do Tocantins, Gurupi 77402-970, TO, Brazil; giongo@uft.edu.br (M.V.G.); rwsa@uft.edu.br (R.W.S.A.); 3 Programa de Pós-Graduação em Biotecnologia, Universidade Federal do Tocantins, Gurupi 77402-970, TO, Brazil; adautoalvessj@gmail.com (A.A.S.J.); thayse.rocha@uft.edu.br (T.C.d.R.); eugenio@ufv.br (E.E.O.); 4 Departamento de Ciências Biológicas, Universidade Estadual de Feira de Santana (UEFS), Bahia, Novo Horizonte 44036-900, BA, Brazil; 5 Programa de Pós-Graduação em Ciências Florestais e Ambientais, Universidade Federal do Tocantins, Gurupi 77402-970, TO, Brazil; 6 Departamento de Agronomia, Universidade Federal Rural de Pernambuco, Recife 52171-900, PE, Brazil; gabriele.160713@gmail.com (A.G.A.); marcos.camara@ufrpe.br (M.P.S.C.); 7 Programa de Pós-Graduação em Biodiversidade e Biotecnologia—Rede Bionorte, Universidade Federal do Tocantins, Palmas 77020-210, TO, Brazil; pimentars@uft.edu.br; 8 Departamento de Entomologia, Universidade Federal de Viçosa, Viçosa, Minas Gerais 36570-900, TO, Brazil

**Keywords:** sclerotia, mycelium, pindaíba, trans-pinocarveol, myrtenal, preventive effect, curative effect

## Abstract

Rice production experiences significant losses due to fungal diseases, particularly rice sheath blight caused by *Rhizoctonia solani*. Despite the intensive and continuous use of synthetic fungicides, diseases severity has not reduced and control has become increasingly challenging; therefore, the search for environmentally friendly and sustainable products has intensified. Here, we conducted a chemical characterization of *Xylopia frutescens* and using in silico analysis evaluated the interaction of their two major compounds with lectin protein site of *R. solani*. *In vitro* tests using increasing concentrations of essential oil against *R. solani* were performed. Subsequently, in four varieties of rice, five concentrations of *X. frutescens* essential oils were applied and evaluated the phytotoxicity effect as well the potential of *Xylopia frutescens* essential oil for controlling, both preventively and curatively, rice sheath blight. We further investigate the selectivity of this essential oil towards the non-target organism, *Trichoderma asperellum*. Our analysis revealed that trans-pinocarveol and myrtenal are the main compounds of *X. frutescens* essential oil and interact with the lectin of *R. solani*, supporting the antifungal properties of *X. frutescens* essential oil. In *in vitro* conditions, the highest tested concentrations of *X. frutescens* essential oil inhibited the pathogen’s sclerotia and mycelial growth. Under greenhouse conditions, the treatments caused low phytotoxicity and effectively reduced disease severity when applied, both preventively and curatively. Furthermore, the biocontrol agent *T. asperellum* exhibited tolerance to *X. frutescens* essential oil. Collectively, our findings demonstrate the potential of *X. frutescens* essential oil for the development of botanical fungicides capable of controlling *R. solani* without harming beneficial non-target organisms such as *T. asperellum*.

## 1. Introduction

To meet the growing demand for agricultural products, high-yield crops have been intensively cultivated worldwide; rice (*Oryza sativa* L.) is the most important of these crops. The global production estimate for the 2024/2025 harvest is 551.5 million tons [1]. In addition to being a high-yield crop, its production is seen as essential to global food security, as it is a key part of the diets of people, especially in Asia and in many countries that need food that is safe, nutritious, and of good quality to keep their populations healthy [2]. However, a key condition for fully realizing the productive potential of these crops is the successful control of diseases. Indeed, phytopathogenic fungi can result in productivity losses of up to 80% [3].

Rice sheath blight, caused by the fungus *Rhizoctonia solani*, is one of the most damaging diseases to the crop [4]. Its incidence occurs from the end of the tillering stage until panicle differentiation during rice cultivation [5]. This pathogen not only attacks the leaf and sheath but also infects rice leaves and panicles, severely compromising productivity and grain quality [6,7]. *R. solani* reproduces through the fragmentation of its mycelium, which can persist for years in the soil as sclerotia. When these resistant structures come into contact with the tissues of a susceptible plant, they have the ability to infect and colonize the new tissue [8]. Once it has infected the plant, *R. solani* can lead to a 50% yield loss in susceptible cultivars under favorable conditions [7,8,9].

At present, the management of diseases has been conducted mainly through synthetic agricultural fungicides as Eprobenfos, Carbendazim, Tebuconazol, Thifluzamide, Propiconazole, Validamycin, Hexaconazole, Pencycuron, methyl thiophanate, and others that are applied as foliar spray or seed treatment [10,11,12]. For rice crops, the fungicide methyl thiophanate is commonly used to control sheath blight caused by *R. solani* [13,14]. Nevertheless, their high and constant utilization has affected efficacy primarily because of the effects on biocontrol agents and the development of resistance in strains of *R. solani* [15,16,17,18,19]. Fungicide resistance in phytopathogens has caused higher dosages and rates of fungicide application, making control ineffective. In addition, the overload of these substances in the environment has had a detrimental impact on human and animal health [18,20,21]. The soil micro fauna can be affected as well. This applies to *Trichoderma asperellum*, which has become popular as a biological control to contain phytopathogens and decrease the occurrence of disease in farm plants [22]. It has also been demonstrated that the *Trichoderma* species may be somewhat tolerant to synthetic fungicides [23]. *Trichoderma* species are unable to work together in controlling fungal diseases because they are sensitive to synthetic fungicides. Thus, the compatibility between any fungicides and the biological control agents, such as the fungus *Trichoderma*, should be evaluated before their combined usage [24,25,26,27].

Natural antifungal products are considered more promising and sustainable alternatives, as far as agriculture is concerned, than traditional fungicides; they have a broad spectrum of biological activity, are persistently low in the environment, and are biodegradable [28,29,30,31,32]. Various studies have demonstrated that plant-derived phytochemicals can exhibit fungicidal effects [33,34,35]. Among these natural compounds, essential oils stand out for being rich in bioactive molecules, such as terpenes, aldehydes, phenols, and alcohols, which, in addition to having antifungal actions, also activate plant defense mechanisms against phytopathogens [36,37,38]. Their mechanisms of action respond to the cell membrane of the fungus, enzymatic activity, and oxidative stress, resulting in cell death [39]. Thus, the search for sources of secondary metabolites in the flora has increased in recent times.

The Cerrado biome is known to have high plant diversity and endemism, and it harbors several natural compounds and essential oils that have a broad spectrum of biological properties [40]. The Annonaceae family is highly diverse, comprising 2106 species across more than 130 genera. Approximately 900 species are Neotropical, 450 are Afrotropical, and the remainder are Indomalayan [41]. This family is present throughout Brazil and is represented in the Cerrado biome by 32 genera and 52 described species [42]. Among these, *Xylopia frutescens Aubl.*, commonly known as “Pindaíba de folha pequena” or “Embira vermelha,” is distributed across all states in the southeastern region, nearly all states in the northern and northeastern regions, as well as in Mato Grosso and Goiás [43]. Despite this diversity, few studies have investigated its fungistatic properties with potential for use in agricultural systems. However, it was reported that essential oils with the major compound of trans-pinocarveol [44,45,46] and that are also major compounds in *X. frutescens* essential oil have antimicrobial activities and other effects, such as larvicidal [47], antitumor, and cytotoxic activities [48,49]. Additionally, this species is native to the Cerrado biome and is very common in the Tocantins state.

Although the given properties of *X. frutescens* essential oil are acknowledged, there is still a serious gap in the body of literature: the literature has not yet conducted any studies on its application against diseases in agricultural plants and its effects on non-target organisms. Thus, the proposed research will explore the possibility of using essential oil extracted from leaves of *X. frutescens* to manage sheath blight, its potential mode of action in the target organism, and its impact on the non-target organism, *Trichoderma asperellum*.

## 2. Results

### 2.1. Chemical Characterization of *Xylopia frutescens* Essential Oil

From 400 g samples of dried leaves, the hydro-distillation process yielded 200 mg of *X. frutescens* essential oil—a yield of 0.20%. Qualitative and quantitative analyses of the components present in the leaf oil of *X. frutescens* (Table 1) revealed a total of 47 constituents. Seven compounds were identified with concentrations exceeding 5% of the total essential oil composition. Among these, trans-pinocarveol (11.49%) was the major constituent, followed by myrtenal (9.99%), α-pinene (7.87%), verbenone (7.16%), myrtenol (6.68%), β-pinene (6.57%), and pinocarvone (6.46%). Thus, several compounds with potential antimicrobial activity were identified in the *X. frutescens* essential oil (Table 1).

### 2.2. Sclerotia Germination and Mycelial Growth of *Rhizoctonia solani* in Response to Increasing Concentrations of *Xylopia frutescens* Essential Oil

Among the treatments evaluated, the essential oil inhibited the germination of pathogen sclerotia starting from a concentration of 7.5 mg/mL, surpassing the effect of the fungicide whose mycelial growth began on the second day (Table 2).

Regarding the mycelial inoculum, only the concentrations of 25 and 50 mg/mL resulted in a significantly high inhibition of mycelial growth, although a small amount of growth was observed starting from the sixth day. In contrast, the fungicide also allowed mycelial growth to begin by the second evaluation day, demonstrating its inefficiency in inhibiting the pathogen (Table 2).

### 2.3. Molecular Docking Study of *Xylopia frutescens* Essential Oil

The molecular docking simulation results indicated that trans-pinocarveol demonstrated the highest binding affinity for the *R. solani* lectin, with a binding energy of −5.36 kcal/mol and an inhibition constant (Ki) of 117.71 nM (Table 3). These results suggest highly favorable interactions and binding stability within the protein’s active site. In contrast, myrtenal yielded a binding energy of −5.01 kcal/mol and a Ki of 213.86 nM, indicating a less stable interaction compared to trans-pinocarveol. The terpenic nature of both compounds is the factor that enabled the establishment of favorable and stable interactions in the active site (Table 3).

**Figure 1:**
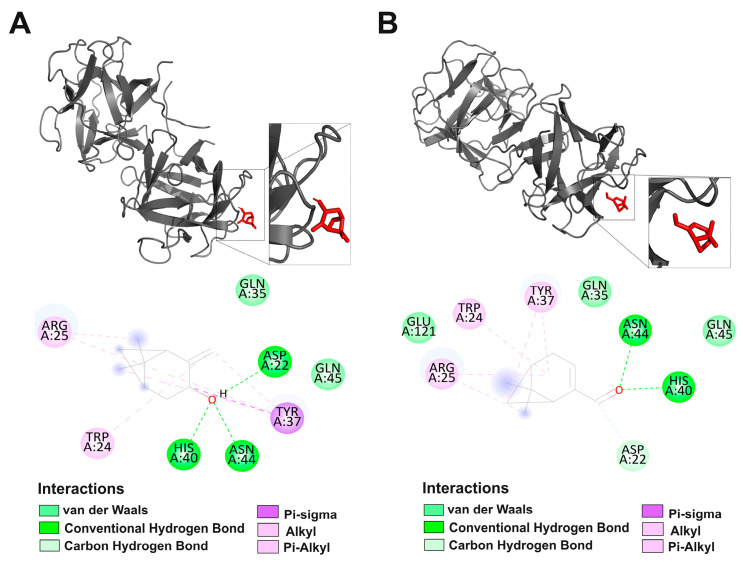
*Rhizoctonia solani* lectin is conjugated to trans-pinocarveol (**A**) and myrtenal (**B**) at the enzyme site. Two-dimensional representations of molecular interactions with amino acids in the protein structures are also provided. The emphasized aspects reveal the molecule’s binding site within the enzyme’s target site.

The simulations revealed that both ligands interacted with the polar agglutinin site of *R. solani*, a region with affinity for N-acetylgalactosamine (GalNAc) (Figure 1A,B) *trans*-pinocarveol established a set of seven interactions with the residues Asp22, Trp24, Arg25, Gln35, Tyr37, His40, and Asn44, forming three hydrogen bonds with Asp22, His40, and Asn44 (Figure 1A). Myrtenal, in turn, exhibited a comparable binding pattern, differing in its interaction with residue Glu121 and in the formation of only two hydrogen bonds with His40 and Asn44 (Figure 1B). Despite the structural differences between the compound groups, an equivalent pattern was observed in the total number of hydrogen bonds: each group of compounds in the essential oil formed a total of five interactions at the active site. However, the monoterpenes (*trans*-pinocarveol and myrtenal) demonstrated a greater selectivity of interaction with specific polar residues of the protein (Figure 1A,B).

### 2.4. Effect of *Xylopia frutescens* Essential Oil on Rice Plant Phytotoxicity

**Figure 2:**
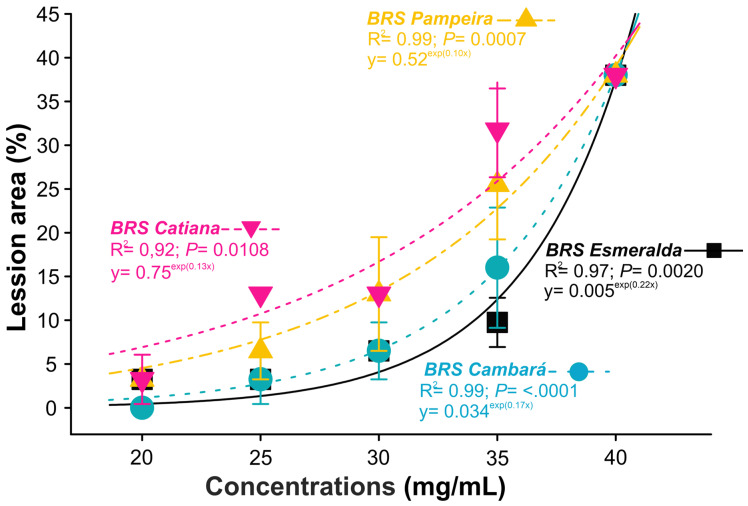
Phytotoxicity of four rice cultivars as a function of the application of different concentrations of *Xylopia frutescens* essential oil, 24 h after exposure. Symbols represent the mean percentage (±SE) of the damaged leaf area.

The results demonstrate that rice plant phytotoxicity was influenced by the cultivar and the concentration of *X. frutescens* oil used. The upland rice cultivars Esmeralda and Cambará exhibited a smaller lesioned leaf area at the lower concentrations of the applied oil. However, the oil applied at 40 mg/mL affected 40% of the rice leaf tissue (Figure 2).

### 2.5. Effect of *Xylopia frutescens* Essential Oil on the Preventive and Curative Control of Sheath Blight Caused by *Rhizoctonia solani*

**Figure 3:**
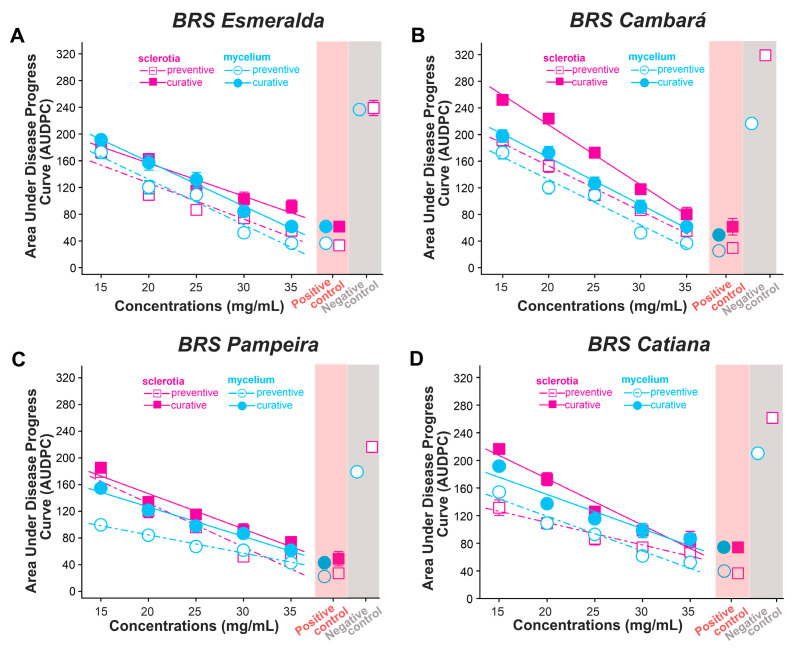
Area Under the Disease Progress Curve (AUDPC) in plants of four rice varieties: Esmeralda (**A**), Cambará (**B**), Pampeira (**C**), and Catiana (**D**) treated with *Xylopia frutescens* essential oil as a preventive or curative measure against inoculation with *Rhizoctonia solani* sclerotia or mycelium. The positive control was methyl thiophanate (20 mg/mL) and negative control was distilled water + Tween 80.

The Area Under the Disease Progress Curve (AUDPC) assessed in the four rice cultivars was significantly reduced after treatment with increasing concentrations of *X. frutescens* essential oil (Figure 3A–D; Table 4). The level of control observed was dependent on the concentration of the essential oil applied either preventively or curatively, the type of pathogen structure inoculated (mycelium or sclerotia), and the resistance of the cultivars used (Table 4). Thus, in the cultivars BRS Cambara and BRS Catiana the AUDPC was lower in preventive treatment when the target was the sclerotia, but in BRS Pampeira the same effect was seen in the mycelium (Table 4). In all cultivars, the AUDPC was markedly reduced at the highest applied concentration, achieving a result similar to the fungicide methyl thiophanate (Figure 3A–D). *X. frutescens* essential oil was more beneficial preventatively, irrespective of the inoculums being sclerotia or mycelium (Figure 3A–D).

### 2.6. Effect of *Xylopia frutescens* Essential Oil on the Non-Target Organism *Trichoderma asperellum*

**Figure 4:**
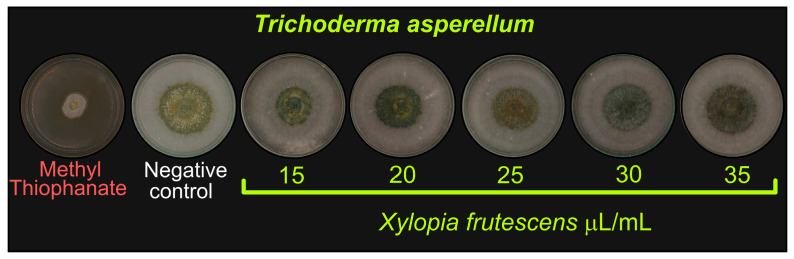
Mycelial growth of *Trichoderma asperellum* after ten days of treatment with different concentrations of *Xylopia frutescens*.

The *X. frutescens* essential oil did not affect *T. asperellum*, including the major concentrations (Figure 4, Table 5). In contrast, the synthetic fungicide showed a strong inhibitory effect, confirming its high toxicity to the biological control agent, as evidenced by high mycelial inhibition (Figure 4, Table 5).

## 3. Discussion

Our results demonstrate that the essential oil of *X. frutescens*, with its high concentration of trans-pinocarveol and myrtenal, effectively inhibited the sheath blight pathogen, *Rhizoctonia solani*, likely through interactions with lectin. Concentrations starting at 35 mg/mL showed low toxic effects on rice leaves, which enabled the reduction in sheath blight when the oil was applied both preventively and curatively. Furthermore, the essential oil exhibited selectivity, showing no deleterious effects on the beneficial fungus *T. asperellum*. This suggests its potential for combined use with *X. frutescens* essential oil as an alternative, sustainable management strategy. This approach could guarantee a reduction in the use of synthetic fungicides for controlling rice sheath blight caused by the etiological agent *R. solani*.

The yield of *X. frutescens* essential oil was lower than that of other studies, which reported a yield of 1.00–1.50% of this species [43,50]. However, it was higher than the 0.15% yield found by Shakri, et al. [51]. As a species of the Annonaceae family, abiotic factors such as nutrition, seasonality, and geographical distribution can influence the essential oil yield in this family [52,53]. As a result of these considerations, in the case of a plant species of specific economic value, it is vital to maximize edaphoclimatic conditions, seasonality, and geographical distribution to enhance the yield of essential oils.

In the analysis of the chemical oil composition, the compounds trans-pinocarveol and myrtenal were found in high quantitative amounts. However, other compounds, such as α-pinene and β-pinene, were also present at significant levels. Other studies have identified α-pinene and β-pinene as the main constituents in the chemical composition of *X. frutescens* [43,54]. Although the present study did not identify them as the major compounds, they still hold quantitative significance. The compounds myrtenol, pinocarvone, and verbenone were also found in the *X. frutescens* essential oil, albeit with low representativeness [43,50]. These differences in composition and compound quantity can also be associated with various biotic or abiotic factors [50,55,56,57].

Trans-pinacarveol and myrtenal are monoterpenes [58], which might be associated with the antifungal property of this essential oil, as they are found in the chemical composition of *X. frutescens* essential oil. The literature proposes a potential relationship between the mechanism of action of the monoterpenes and the induction of microbial membrane disruption. This can be attributed to the fact that these chemical compounds can bind to ergosterol, consequently forming channels and hence increasing fluidity and permeability and thus the destabilization of the fungal cell membranes. This action can be linked to their non-polar nature, which weakens the lipid structure of fungi [59]. These compounds demonstrated an interaction with the lectin site of *R. solani*, highlighting their promise in exerting antifungal activity against the causal agent of sheath blight. This interaction is due to the fact that the lectin accumulates in the mycelium and sclerotia of the soil-borne phytopathogenic fungus *R. solani*. Although it is considered a storage protein, it is implied that the fungicidal activity against the fungus is due to the interaction with this protein [60].

In vitro assays for *X. frutescens* essential oil are scarce; however, our results demonstrated toxic activity against the germination of sclerotia and the mycelium of *R. solani*. The antifungal potential of essential oil has been reported in related species: For the fruits of *Xylopia aethiopica* used against *Aspergillus niger* and *Fusarium oxysporum*, a minimum inhibitory concentration (MIC) of 3 mg/mL of the oil was found *in vitro* [61]. In another study, *Candida albicans* was characterized as an essential oil of *X. aethiopica* with an MIC of 50 mg/mL [62]. Conversely, in the present experiment, where *X. frutescens* leaf essential oil and *R. solani* were used, the inhibitory effect was seen at 10 mg/mL concerning sclerotia and at 25 mg/mL in the case of mycelium. These are higher concentrations compared to the MIC presented by Tegang, Beumo, Dongmo, and Ngoune [61]. Nevertheless, the results obtained in the present study were superior when compared to [62].

Another study reported an 87.63% mycelial inhibition of *Sclerotinia sclerotiorum* using 300 mg of essential oil extracted from the leaves of *Cardiopetalum calophyllum*, a species belonging to the same family as *X. frutescens*, the Annonaceae [63]. The mycelial inhibition of *R. solani* in this study at a concentration of 50 mg/mL (86.68%) was relatively close to that found by these authors. Similar to *S. sclerotiorum*, the fungus *R. solani* is an important soil-borne phytopathogen that also forms resistant structures known as sclerotia. The results demonstrate that in controlling these structures, *X. frutescens* oil was more effective, with a concentration of 7.5 mg/mL inhibiting 93.33% of *R. solani* sclerotia germination. At concentrations above 7.5 mg/mL, 100% inhibition was achieved. The presence of *R. solani* mycelial growth on the sixth day of incubation with concentrations of 25 and 50 mg/mL can be explained by the volatility of the bioactive compounds that form in the essential oil and are believed to be the source of the antifungal activity [64]. According to the results, the inhibition of the growth of *R. solani* sclerotia and mycelium in the *in vitro* experiment by monoterpenes such as trans-pinocarveol and myrtenal, the essential oils in *X. frutescens*, could be caused by the terpenes that are known to affect cellular respiration [50,65].

The *X. frutescens* essential oil studied here resulted in mild damage to rice leaves. This phytotoxic activity on the leaves was probably explained by the presence of terpenes as one of the chemical compounds in the essential oil [66]. Moreover, the induction of defense mechanisms against phytopathogens in agricultural plants with particular concentrations of *X. frutescens* essential oil was proved by another study [38].

In studies that involved the genus *Xylopia* spp., there were few documents of bioassays performed on the phytotoxicity of the plants, and thus it was necessary that further studies be conducted on the same to establish the right concentration that could be used on crops. The reason is that the majority of studies were conducted only through *in vitro* testing [67,68]. Thus, it is critical to set the procedures, which are established by bioassays, to establish the highest concentration acceptable by any cultivar. Accurate oil concentrations are also significant to illustrate the economic feasibility of their application to farming [69,70]. Furthermore, toxicity tests can be carried out because it is essential to ensure that the concentrations are potent against the target organisms and have no detrimental impact on the plant [71].

Our results demonstrated the antifungal activity of *X. frutescens* essential oil against *R. solani* in both *in vitro* assays and in preventive and curative trials, where it showed high efficacy in controlling rice sheath blight. A review of the literature reveals limited studies on the application of essential oils from *X. frutescens* or the *Xylopia* genus in agriculture. Additionally, we also found that the resistance of the rice cultivars was influenced by the type of pathogen inoculum (whether by sclerotia or mycelium of *R. solani*), as well as by the preventive or curative application of *X. frutescens* essential oil in controlling sheath blight. Inoculation with sclerotia resulted in lower disease severity across all rice cultivars when the oil was applied preventively. It should also be considered that cultivars may exhibit different levels of resistance to sheath blight due to possessing multiple metabolic pathways, including the metabolism of amino acids, carbohydrates, cofactors, and vitamins, as well as terpenoid and polyketide metabolism [5,72,73,74]. Assuming that essential oils contain terpene compounds in their chemical composition, they may confer greater resistance in conjunction with the factors mentioned above, allowing for enhanced protection against fungal pathogens [75,76].

In addition to the potential use of *X. frutescens* for controlling diseases in agricultural plants like rice, further research should explore its efficacy in other pathosystems. It is known that sclerotia, being the primary form of contact with rice plants in the field, enable the initial infection of rice tissues, causing sheath blight [77,78,79]. Therefore, the protection of plant tissues by *X. frutescens* essential oil against both types of infectious structures (sclerotia and mycelium) used in this work is of great importance. Evidence of this importance has already been found in another recent study, which demonstrated the use of *X. frutescens* essential oil to control a disease also caused by *R. solani* in cowpea, as well as to control leaf spots in maize caused by another pathogen, *Curvularia lunata* [38]. Future studies should focus on other pathosystems and pathogen infections in plants, including spore-reproducing fungi.

This study demonstrated that *Trichoderma asperellum* was not inhibited by the application of increasing concentrations of *X. frutescens* essential oil. On the other hand, *T. asperellum* has previously demonstrated its antagonistic potential against *R. solani* infection in rice plants, protecting against sheath blight [80,81]. Thus, the use of *X. frutescens* essential oil does not practically influence *T. asperellum* and can, therefore, be implemented as a part of the integrated disease management of rice. This indicates the potential for its combined use with this biological control agent to manage sheath blight in rice cultivation. Further studies are required to examine the use of the two agents together, as well as the use of other synthetic fungicides and control methods to minimize the intensity of sheath blight in rice plants without impacting the non-target organisms.

## 4. Materials and Methods

### 4.1. Pathogen Isolation

The fungus *R. solani* was isolated from irrigated rice crops at the Cooperativa Agroindustrial Rio Formoso Ltd. (Cooperformoso), located in Formoso do Araguaia, Tocantins, Brazil (latitude 12°00′07″ S and longitude 49°40′05″ W). *R. solani* isolates were obtained from diseased plants of the Pampeira cultivar, exhibiting typical symptoms of sheath burn. In the phytopathology laboratory at the Federal University of Tocantins (UFT), small damaged fragments were removed from the infected sheaths; they were disinfected with alcohol (50%) for 30 s, followed by sodium hypochlorite (1%) for 40 s. The cultures were then moved to Petri dishes with the potato dextrose agar (PDA) medium. The plates were incubated at 25 ± 2 °C under a 12 h photoperiod. The fungus *R. solani* remains stored in the mycotheca of the phytopathology laboratory at the Federal University of Tocantins (UFT, Gurupi, TO, Brazil), with isolation code UFT-Rs:13.

### 4.2. Collection, Extraction, and Chemical Characterization of *Xylopia frutescens* Essential Oil

Leaves of *X. frutescens* were collected in Agroindustrial Rio Formoso Ltd. Cooperformoso, Formoso do Araguaia, Tocantins, Brazil (11°15′59″ S, 49°43′43″ W, altitude 235 m) and transported according to the method described by Seixas, et al. [82], with adjustments for essential oil extraction.

The essential oil was extracted by hydro-distillation using a modified Clevenger apparatus, following the methodology of Guimarães, et al. [83]. The leaves were air-dried at room temperature for 5 days and then ground in a Wiley knife mill. The process of hydro-distillation of oil in 400 g of ground in 1 L of distilled water in the Clevenger-type apparatus took 2 h. The extract was then transferred to an amber vial containing the supernatant oil that was placed under the conditions of 4 °C until use in the assays. A net essential oil yield of 200 microliters (0.20%) was obtained.

The analysis of the essential oil sample was performed by gas chromatography coupled with mass spectrometry (GC-MS), as described by Osorio, et al. [84]; the essential oil yield was calculated based on the dry plant mass and expressed as mL/100 g of dry material, following the formula [essential oil extracted (mL)/initial mass dry plant (g)] × 100 as proposed by Cascaes, Marques da Silva, de Oliveira, Cruz, de Moraes, do Nascimento, Ferreira, Guilhon, and Andrade [50].

### 4.3. *Antifungal Xylopia* frutescens Essential Oil Antifungal Potential Against *Rhizoctonia solani*

In vitro bioassays were conducted using Petri dishes (90 mm in diameter) containing PDA culture medium. Initially, a stock solution of the highest concentration of essential oil (50.0 mg/mL) mixed with Tween 80 (2 mg/mL) and distilled water was prepared in a 10 mL volumetric flask. The flask was gently shaken to obtain a homogeneous mixture. Subsequently, dilutions were performed until the other concentrations of 5.0, 7.5, 10.0, and 25.0 mg/mL were obtained. Then, 200 μL of each concentration was uniformly distributed on the surface of the culture medium using the Drigalsky^®^ spatula.

Each Petri dish was inoculated with a 6 mm diameter PDA disk, which contained mycelium or sclerotia from *R. solani.* The plates were covered, marked, and incubated at 25 ± 2 °C with 12/12 h photoperiod for ten days. Four replicates were involved in each treatment. The mycelial growth was measured, and the colony diameter was measured every two days; a total of five measurements were taken. Two opposite points were used to determine the average diameter using a digital caliper. The treatments used were a positive control (20 mg/mL of methyl thiophanate) and a negative control (PDA containing a solution of sterile distilled water and Tween 80). The percentage of mycelial growth inhibition (MGI) was calculated from the measurements according to the formula described by Krutmuang, et al. [85].

### 4.4. *Xylopia frutescens* Essential Oil Molecular Docking

The two most significant (major) compounds in the *X. frutescens* essential oil, trans-pinocarveol and myrtenal, were chosen to be studied in silico. The PubChem Compound cheminformatics database (RCSB PDB, https://www.rcsb.org, access on 5 December 2025) was used to obtain the tested compounds. The files were received in the format of ‘.sdf’ and transformed to the format of ‘.pdb’ with the help of PyMol 3.0 [86,87].

Based on the affinity of essential oil compounds for the *R. solani* lectin [88], molecular docking simulations were performed using the crystallographic three-dimensional structure of the protein (PDB: 4G9N) [60], which was downloaded from the Protein Data Bank (RCSB PDB) (Schrodinger, LLC). The file was obtained in ‘.pdb’ format, and the software PyMol 3.0.4 (Schrodinger, LLC) was used to remove water molecules, ions, and co-crystallized ligands from the protein file.

The receptor and ligands were prepared for molecular docking using AutoDock Tools 1.5.7. Hydrogen atoms were included to compute protonation states, and all possible bond torsions were computed on the ligands. The grid box (40 × 40 × 40 Å) was then constructed inside the receptor, giving the locations where the ligands would be docked into the GalNAc binding site of chain A (ASP22, HIS40, ASN44, ARG25, GLN35, GLU121, TYR37, and TRP24) [60].

The receptors and ligands were then saved in the ‘.pdbqt’ format. The software AutoDock 4.2 was used to generate 100 docking poses for each ligand interacting with the protein’s active site, thereby obtaining binding affinity values (kcal/mol) and inhibition constants (nM). The results were interpreted by analyzing the positioning of the ligands in the precise active site region, and interaction maps were generated using PyMol 3.0.4 (Schrodinger, LLC) and Discovery Studio Visualizer (v21.1.0.20298) [89,90].

### 4.5. Phytotoxicity of *Xylopia frutescens* Essential Oil on Rice Plants

The essential oil toxicity assay was conducted under greenhouse conditions (27 ± 5 °C; 80% relative humidity; 12:12 light/dark). The experimental design was completely randomized, in a factorial scheme, with four rice cultivars and an essential oil applied. Four rice cultivars were used for planting: two recommended for upland cropping systems (BRS Esmeralda and BRS Cambará) and two for the irrigated system (BRS Pampeira and BRS Catiana). The plants were grown in 11 L polyethylene pots (25 cm in height, 28 cm top diameter, and 22 cm base diameter) filled with a substrate composed of soil amended with 10 g of NPK fertilizer (5-25-15), 300 g of manure, 10 g of zinc sulfate, and 20 g of limestone per pot. Ten seeds were sown per pot and subjected to daily irrigation. Fifteen days after planting, thinning was performed, leaving only four plants per pot. A water depth of 2.5 cm was maintained for irrigated rice cultivars after thinning to ensure plant tillering. Thirty days after plant emergence, manual spray application of 5 mL per pot of the five *X. frutescens* essential oil concentrations (20, 25, 30, 35, and 40 mg/mL) was carried out, with four replicates. These doses were predetermined based on the *in vitro* results. Phytotoxicity assessments were performed 24 h after application. Essential oil toxicity was measured using a scoring scale used in a number of studies [91,92,93] on the presence or absence of chlorosis or necrosis symptoms on the plants or leaves.

### 4.6. Use of *Xylopia frutescens* Essential Oil in the Preventive and Curative Control of Rice Sheath Blight

Based on the *in vitro* and phytotoxicity results, preventive and curative trials for the control of sheath blight were established with infections caused by sclerotia and mycelial inocula of *R. solani*. The experimental conditions, including the greenhouse environment, pot substrate composition, and plant development stage (30 days after emergence), were the same as in the phytotoxicity assay for both trials. The four rice cultivars (as presented in the phytotoxicity assay above) were taken, mixed with five levels of essential oil (15, 20, 25, 30, and 35 mg/mL), and two inoculations of *R. solani* (mycelium and sclerotia) with four replicas per treatment. A positive control (20 mg/mL of methyl thiophanate) and a negative control (diseased plants with *R. solani* inoculum) were included.

For the preventive assay, 5 mL of the essential oil solution was first sprayed onto the rice plants. After a period of 2 h, two mycelial disks (6 mm diameter) and sclerotia of *R. solani* were inoculated at the base of the leaf sheath (crown), near the soil surface, for each replicate. The inoculated plants were transferred and maintained in humidity chambers for 48 h. The plants were then incubated in a controlled-temperature room (25 ± 2 °C) over a period of 10 days, whereby the severity of the disease was measured after every two days until a total of five measurements were made. The severity of the disease was determined on the leaves and sheaths using the adapted scale that was reported by Santos, et al. [94], and was on a scale of 0 to 9. This scale describes the percentage of tissue affected by disease in six levels, with 0 being healthy plants and 9 being more than 50% of the area having symptoms. The cause of the infection by both sclerotia and mycelium of *R. solani* was calculated using the Area Under the Disease Progress Curve (AUDPC) of the severity data using the formula presented by Schneider, et al. [95].

The curative control assay maintained the same methodology as the preventive control (including the assessments). However, in this case, inoculation with *R. solani* was performed before the application of the essential oil. Three days after inoculation, following the appearance of the first lesions on the rice plants, the same essential oil solutions were applied.

### 4.7. Assay of the Effect of *Xylopia frutescens* on *Trichoderma asperellum*

For the non-target organism selectivity assay, the concentrations used were 15, 20, 25, 30, and 35 mg/mL of *X. frutescens* essential oil, which were the same as those applied in the preventive and curative treatments. The fungus *T. asperellum* was obtained from the culture collection of the plant pathology laboratory at the Federal University of Tocantins (UFT, Gurupi, TO, Brazil). Initially, *T. asperellum* was cultured on potato dextrose agar (PDA). Two days after inoculation, the essential oil solutions (15, 20, 25, 30, and 35 mg/mL) were sprayed onto the surface of the medium. Sterile distilled water with Tween 80 polysorbate and methyl thiophanate were designated as the negative and positive controls, respectively. Mycelial growth was measured every two days until the tenth day in all treatments. Based on the assessments, the percentage of mycelial growth inhibition (MGI) was calculated using the formula proposed by Krutmuang, Rajula, Pittarate, Chatima, Thungrabeab, Mekchay, and Senthil-Nathan [85].

### 4.8. Statistical Analysis

The data obtained from the phytotoxicity bioassays and the AUDPC values were subjected to nonlinear and linear regression, respectively, using SigmaPlot 12.5. To verify if the two regressions differed from each other, we analyzed the main effects of group and X, in addition to the group × X interaction term. The significance of the interaction was used to determine if the slopes differed between groups using the PROC GLM procedure using SAS 9.1 software [96].

## 5. Conclusions

Our findings highlight the potential of *Xylopia frutescens* essential oil to control rice sheath blight under both inoculation conditions. Our findings further reveal differences in cultivar resistance depending on whether sclerotia or mycelium were used as an inoculum source. We identified numerous chemical compounds that may be associated with the antifungal activity of *X. frutescens* essential oil, with emphasis on the major compounds. Molecular docking supported these findings by confirming the interaction of the trans-pinocarveol and myrtenal with the lectin protein of *R. solani*. Furthermore, the concentrations applied in the preventive and curative assays showed no toxic effects on *T. asperellum*, indicating selectivity toward the non-target beneficial organism. Although the use of essential oils with fungicidal effects does not seem to be economically viable for large-scale crops; their use in integrated disease management in small-scale crops may be viable and safe.

## Figures and Tables

**Table 1:** Chemical constituents of *Xylopia frutescens* essential oil identified by GC/MS and their respective concentrations expressed as a percentage.

CN	Constituents	RT	RIC	RIR	%
1	2,5-cyclohexadiene-1-methanol	3890	3860	3925	0.29
2	α-pinene	4969	4915	5105	7.87
3	Thuja-2,4(10)-diene	5430	5380	5525	2.05
4	β-pinene	5948	5890	6070	6.57
5	o-Cymol	7160	7110	7210	2.06
6	Eucalyptol	7305	7210	7415	2.53
7	Comphene	7475	7415	7547.5	1.62
8	Canfenilone	8690	8640	8755	0.39
9	Dihydrocarveol	8855	8785	8880	0.51
10	α-campholenal	8900	8880	8960	0.38
11	α-fenchocamphorona	9261	9230	9300	0.39
12	Phenylic alcohol	9544	9490	9595	0.61
13	α-fluorohenaldehyde	9862	9815	9925	1.34
14	Nopinone	10,126	10,065	10,170	3.74
15	Trans-pinocarveol	10,219	10,170	10,275	11.49
16	Cis-verbenol	10,299	10,275	10,360	1.21
17	Trans-verbenol	10,401	10,360	10,460	3.07
18	Mentha-1,5-dieno-8-ol	10,506	10,460	10,610	1.23
19	2-methylene-6,6-dimethyl-bicyclo [3.2.0] heptane-3-ol	10,727	10,670	10,760	0.69
20	Pinocamphone	10,785	10,760	10,805	0.46
21	Pinocarvone	10,845	10,805	10,900	6.46
22	3,5-Dimethyl-5-ethyl-. DELTA [2]-pyrazoline	10,915	10,900	10,940	0.45
23	Borneol	10,980	10,940	11,000	1.32
24	p-1,5-Menthodienol-8	11,028	11,000	11,130	2.61
25	(2,2,6-trimethyl-bicyclo[4.1.0]hept-1-yl)-methanol	11,221	11,130	11,255	0.5
26	4-terpineol	11,295	11,255	11,345	0.95
27	Mirta	11,394	11,345	11,485	0.76
28	P-cymen-8-ol	11,557	11,490	11,640	1.37
29	α-TERPINEOL	11,687	11,640	11,725	0.78
30	Myrtenal	11,777	11,725	11,810	9.99
31	Myrtenol	11,839	11,810	11,980	6.68
32	Verbenone	12,139	12,085	12,280	7.16
33	Cis-carveol	12,475	12,425	12,560	1.03
34	Cyclooctene, 3-(1-methylethenyl)	13,940	13,895	14,015	0.39
35	Perillyl alcohol	14,641	14,600	16,685	0.32
36	Cycloactive	16,370.5	16,332.5	16,417.5	0.7
37	Copaene	16,625	16,580	16,685	0.43
38	Thujpsadiene	17,380	17,335	17,430	0.29
39	2,3,3-Trimethyl-2-(3-methyl-buta-1,3-dienyl)-cyclohexanone	20,978	20,930	21,020	0.44
40	1H-cycloprop[e]azulen-7-ol, decahydro-1,1,7-trimethyl-4-methylene-, [1ar-(1a.α,4a.α,7 β,7a. β,7b.α)]	21,577	21,470	21,655	4.33
41	Caryophyllene oxide	21,682	21,655	21,765	0.7
42	Spiro[4.5]dec-6-en-8-one, 1,7-dimethyl-4-(1-methylethyl)	22,191	22,135	22,225	0.28
43	4,8,8-trimethyl-2-methylene-bicyclo[5.2.0]nonane-	22,299	22,225	22,350	0.43
44	Isospathulenol	22,761	22,705	22,860	0.75
45	Widdrol	23,290	23,235	23,345	0.92
46	(4,6,8,9-tetramethyl-3-oxabicyclo[3.3.1]non-6-eno-1-yl)methyl acetate	23,390	23,345	23,475	0.72
47	Mustakone	23,831	23,770	23,880	0.74
Total	-	600,460	598,143	605,510	100

CN: compound number; RT: retention time; RIC: calculated retention indices; RIR: literature retention index; %: relative abundance.

**Table 2:** Effect of *Xylopia frutescens* essential oil in the germination (sclerotia) and growth (mycelial) of *Rhizoctonia solani*.

Treatment (mg/mL)	Sclerotia Germination Over Time (mm ±SE)					IGS ^a^ (%)	Mycelial Growth Over Time (mm ± SE)					IGM ^b^ (%)
	2	4	6	8	10		2	4	6	8	10	
Control	78.2 ± 9.5	90 ± 0	90 ± 0	90 ± 0	90 ± 0	-	42.6 ± 1.9	90 ± 0	90 ± 0	90 ± 0	90 ± 0	-
Methyl thiophanate (20)	12 ± 0.4	13.5 ± 0.6	13.5 ± 0.6	13.5 ± 0.6	13.5 ± 0.6	85	8.1 ± 3.4	18 ± 3.8	18.3 ± 6.3	18.3 ± 6.3	18.3 ± 6.3	79.95
5.0	-	47.7 ± 8.2	90 ± 0	90 ± 0	90 ± 0	29.4	18.6 ± 1.7	90 ± 0	90 ± 0	90 ± 0	90 ± 0	11.3
7.5	-	-	-	-	30 ± 25	93.3	16.2 ± 0.4	86.1 ± 3.2	90 ± 0	90 ± 0	90 ± 0	13.3
10.0	-	-	-	-	-	100	12 ± 0.8	76.4 ± 0.8	90 ± 0	90 ± 0	90 ± 0	17.4
25.0	-	-	-	-	-	100	-	-	23 ± 10.6	54.2 ± 22.5	60 ± 24.5	70.8
50.0	-	-	-	-	-	100	-	-	7.3 ± 6	26.3 ± 21.5	30 ± 24.5	86.7

^a^ Inhibition germination sclerotia; ^b^ inhibition growth mycelium.

**Table 3:** Molecular docking for the complexes between the major compounds of *Xylopia frutescens* and the *Rhizoctonia solani* lectin.

Specie	Molecule	Bond Energy (Kcal/mol)	Ki (nM)
*Xylopia frutescens*	Trans-pinocarveol	−5.36	117.71
	Myrtenal	−5.01	213.86

**Table 4:** Summary of the linear regression analyses showed in Figure 3A–D.

Model	Cultivate	Target	Exposure	Estimated Parameters (±SE)		df Error	*F*	*P*	*R^2^*
				a	y0				
f = y0 + ax	BRS Esmeralda	sclerotia	preventive	−5.40 ± 1.12 a	234.50 ± 29.13 a	4	23.18	0.0171	0.88
			curative	−4.95 ± 0.75 a	255.25 ± 19.68 a	4	42.67	0.0073	0.93
		mycelium	preventive	−6.81 ± 0.80 a	268.43 ± 20.95 a	4	71.36	0.0035	0.94
			curative	−6.66 ± 0.38 a	291.68 ± 10.06 a	4	296.02	0.0004	0.99
	BRS Cambará	sclerotia	preventive	−6.77 ± 0.43 a	288.37 ± 1.24 a	4	245.07	0.0006	0.98
			curative	−9.00 ± 0.51 b	394.38 ± 13.45 b	4	301.86	0.0004	0.99
		mycelium	preventive	−6.81 ± 0.80 a	268.43 ± 20.95 a	4	71.36	0.0035	0.96
			curative	−7.07 ± 0.34 a	306.87 ± 9.02 b	4	414.96	0.0003	0.99
	BRS Pampeira	sclerotia	preventive	−6.56 ± 1.02 a	263.31 ± 26.69 a	4	40.79	0.0078	0.93
			curative	−5.30 ± 0.71 a	252.50 ± 18.53 a	4	55.16	0.0051	0.95
		mycelium	preventive	−2.72 ± 0.21 a	139.13 ± 5.63 a	4	157.71	0.0011	0.98
			curative	−4.42 ± 0.40 b	215.00 ± 10.60 b	4	117.63	0.0017	0.98
	BRS Catiana	sclerotia	preventive	−3.25 ± 0.42 a	175.00 ± 11.04 a	4	58.41	0.0047	0.95
			curative	−6.73 ± 0.82 b	308.19 ± 21.33 b	4	67.31	0.0038	0.95
		mycelium	preventive	−5.02 ± 0.65 a	219.50 ± 17.06 a	4	58.52	0.0046	0.95
			curative	−4.97 ± 0.94 a	250.13 ± 24.46 a	4	192.23	0.0007	0.90

Identical lowercase letters in “parameter a” and on the same target in two exposure measurements mean there is no statistically significant difference in the response variable between the two groups (X × group); a similar representation is shown for the “parameter y0” (intercept) PROC GLM (SAS, 9.1).

**Table 5:** Effect of *Xylopia frutescens* essential oil on the mycelial growth inhibition (MGI) of *Trichoderma asperellum*.

Treatments (mg/mL)	Mycelial Growth Over Time (mm ± SE)					MGI
	2	4	6	8	10	(%)
Control	55.8 ± 1.06	90 ± 0	90 ± 0	90 ± 0	90 ± 0	0.00
Methyl thiophanate	17.73 ± 1.05	17.73 ± 0.94	17.73 ± 0.94	17.73 ± 0.94	17.73 ± 0.94	77.88
15.0	53.32 ± 2.19	90 ± 00	90 ± 00	90 ± 00	90 ± 00	1
20.0	49.23 ± 1.20	90 ± 00	90 ± 00	90 ± 00	90 ± 00	2
25.0	46.61 ± 1.26	90 ± 00	90 ± 00	90 ± 00	90 ± 00	3
30.0	46.61 ± 1.26	90 ± 00	90 ± 00	90 ± 00	90 ± 00	3
35.0	39.97 ± 1.22	90 ± 00	90 ± 00	90 ± 00	90 ± 00	6

## Data Availability

The original contributions presented in this study are included in the article. Further inquiries can be directed to the corresponding author.

## Abbreviations

The following abbreviations are used in this manuscript:

GC-MS	Gas chromatography coupled with mass spectrometry
GalNAc	N-acetylgalactosamine
